# Differential Effects of Green Space Typologies on Congenital Anomalies: Data from the Korean National Health Insurance Service (2008–2013)

**DOI:** 10.3390/healthcare13151886

**Published:** 2025-08-01

**Authors:** Ji-Eun Lee, Kyung-Shin Lee, Youn-Hee Lim, Soontae Kim, Nami Lee, Yun-Chul Hong

**Affiliations:** 1 Department of Human Systems Medicine, College of Medicine, Seoul National University, Seoul 03080, Republic of Korea; leje93@snu.ac.kr (J.-E.L.); 65855@snuh.org (N.L.); 2 Center for Public Healthcare Policy, National Medical Center, Seoul 04564, Republic of Korea; kslee0116@nmc.or.kr; 3 Section of Environmental Health, Department of Public Health, University of Copenhagen, 1014 Copenhagen, Denmark; limyounhee@gmail.com; 4 Department of Environmental and Safety Engineering, Ajou University, Suwon 16499, Republic of Korea; soontaekim@ajou.ac.kr; 5 Department of Public Health, Seoul National University Hospital, Seoul 03080, Republic of Korea; 6 Institute of Environmental Medicine, Seoul National University Medical Research Center, Seoul 03080, Republic of Korea

**Keywords:** built environment, case–control study design, congenital anomalies, green space

## Abstract

**Background/Objectives**: Urban green space has been increasingly recognized as a determinant of maternal and child health. This study investigated the association between prenatal exposure to different types of green space and the risk of congenital anomalies in South Korea. **Methods**: We analyzed data from the National Health Insurance Service (N = 142,422). Green space exposure was measured at the area level and categorized into grassland and forest; statistical analysis was performed using generalized estimating equations and generalized additive models to analyze the associations. Additionally, subgroup and sensitivity analyses were performed. **Results**: GEE analysis showed that a 10% increase in the proportion of grassland in a residential district was associated with a reduced risk of nervous system (adjusted odds ratio [aOR]: 0.77, 95% confidence interval [CI]: 0.63–0.94) and genitourinary system anomalies (aOR: 0.83, 95% CI: 0.71–0.97). The subgroup analysis results showed significance only for male infants, but the difference between the sexes was not significant. In the quartile-based analysis, we found a slightly significant *p*-value for trend for the effect of forests on digestive system anomalies, but the trend was toward increasing risk. In a sensitivity analysis with different exposure classifications, the overall and nervous system anomalies in built green space showed that the risk decreased as green space increased compared to that in the lowest quartile. **Conclusions**: Our results highlight the importance of spatial environmental factors during pregnancy and suggest that different types of green spaces differentially impact the offspring’s early health outcomes. This study suggests the need for built environment planning as part of preventive maternal and child health strategies.

## 1. Introduction

According to the World Health Organization, approximately 6% of newborns are born with congenital anomalies (CAs) worldwide. These CAs contribute to a considerable fraction of infant mortality, with an estimated 17–43% of infant deaths worldwide [1]. In South Korea, the prevalence of major CA among newborns born in 2013–2014 was 433.5 per 10,000 children, and the total mortality was 1.7% [2]. As some CAs can cause long-term disability and have a negative impact on the child’s quality of life [3], many studies have attempted to identify the underlying causes of these conditions [4,5].

Recent studies has highlighted the influence of genetic and environmental factors on CAs, and environmental risks associated with CAs include exposure to air pollution [6], extreme heat [7,8], and heavy metals during pregnancy [9]. Urban green spaces provide residents with several benefits including improved air quality and climate regulation [10], increased social connectivity in open spaces, and promotion of physical activity [11], indicating potential protective effects on fetal and maternal health. However, despite these potential protective mechanisms, studies specifically examining the association between green space exposure and congenital anomalies remain very limited. A Chinese study found that increased green space was associated with reduced congenital heart defects [12], while a US study found that residential green space lowered the risk of certain structural defects [13]. Nevertheless, the current evidence is limited and inconclusive, highlighting a significant research gap in understanding how prenatal green space exposure affects CA risk.

An increasing body of evidence suggests that health benefits of green spaces depend not only on quantity but also qualitative characteristics such as accessibility, composition, and integration with urban environments. Urban green spaces can vary significantly in ecological characteristics and plant diversity, accessibility and use patterns, resulting in different health outcomes [14]. Studies have demonstrated that green space morphology, particularly higher aggregation and connectivity, was associated with better health outcomes [15], while accessible street trees showed different effects compared to remote natural areas [16]. Research on green space morphology and non-communicable diseases found that more connected, aggregated, and complex-shaped green space configurations provided greater health benefits [17]. These findings suggest that nature interactions and accessibility patterns mediate differential exposure effects [18], indicating that the type and accessibility of green space may be more important than merely the vegetation quantity. Therefore, this study aimed to fill the research gap by analyzing the differential health effects of green space on CAs according to accessibility in South Korea.

## 2. Materials and Methods

### 2.1. Data and Variables

This study used National Health Insurance Service (NHIS) claim data of children born from 2008 to 2013 in South Korea. Potential participants were identified using International Classification of Diseases—10th Revision (ICD-10) codes. Those with a record of the following congenital diseases from birth to 6 years of age were included in the “case” group: congenital anomalies of the nervous system (Q01–Q05); of the eye, ear, nose, face, and neck (Q11–Q17, Q30, and Q35–Q37); circulatory system (Q20–Q26); digestive system (Q39–Q45); genitourinary system (Q53, Q54, Q56, Q60–Q62, and Q64); musculoskeletal system (Q65, Q66, Q69–Q75, Q77, and Q79); and overall CA group (Q01–Q79).

Those who visited a hospital before 6 years of age for noninfectious gastroenteritis and colitis (ICD-10: K50–K52) and never visited a hospital for a CA were included in the “control” group. Noninfectious gastroenteritis and colitis were selected in light of several considerations. First, these conditions are common pediatric diseases with well-defined diagnostic criteria [19]. Second, unlike respiratory conditions that benefit from green spaces through multiple pathways including air quality improvements, noninfectious gastroenteritis and colitis are unlikely to be influenced by the primary mechanisms through which green spaces confer health benefits [20]. Third, in contrast to structural CA which originate from developmental disruptions during embryogenesis, these functional gastrointestinal conditions are primarily shaped by postnatal factors such as diet, infections, and microbiome development [21]. Fourth, these conditions occur across all socioeconomic strata, minimizing selection bias [22]. In addition, controls were selected from among children with at least one hospital visit to ensure comparable healthcare access and minimize detection bias.

Controls were matched to cases at a 3:1 ratio based on place of birth (city or province), birth year, sex, delivery type, and policyholder’s income level. A total of 1,444,207 claims were identified between 1 January 2008 and 31 December 2015. Of the total claims, 998,985 were excluded owing to death (*n* = 2953), unknown date of delivery (*n* = 294,253), premature birth (*n* = 60,906), or endocrine anomalies (*n* = 640,873). Based on the purpose of the study, 244,402 claims in rural areas, 380 claims in Ganghwa County near the military demarcation line, and 58,018 claims in 2014 and 2015 were also excluded. Therefore, a total of 142,422 people were included in the study (Figure S1). The institutional review board of the Seoul National University Hospital, Republic of Korea, exempted this study from ethical review because the data were de-identified (Institutional Review Board No. 1702-047-830).

### 2.2. Exposure to Green Space

In this study, we used land cover maps that can classify green space typologies. Many researchers use normalized difference vegetation index (NDVI), which defines the degree of greenness on a scale of −1 to +1, but this study utilized land cover maps to identify differences according to green space typologies. Data on green spaces accessible during pregnancy were classified according to standardized satellite images of land cover maps with a resolution of 0.25 m, which were provided by the Environmental Geographic Information Institute under the Ministry of Environment in South Korea. Land cover maps were classified into Level-1 (resolution: 30 m), Level-2 (resolution: 5 m), and Level-3 (resolution: 1 m), and this study used the high-resolution Level-3 map to accurately identify detailed green space in urban areas. We distinguished between forest and grassland based on accessibility patterns in the South Korean urban context. Forests, predominantly located in mountainous areas, require active travel and may be less accessible to pregnant women, while grasslands include neighborhood integrated green spaces (street trees, apartment landscaping, local parks) that provide continuous exposure opportunities throughout daily activity.

**Figure 1:**
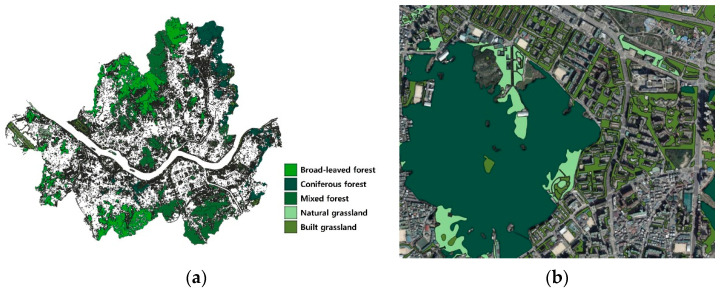
Land cover map for different vegetation types. (**a**) Spatial distribution of different types of urban green spaces in Seoul, categorized into broad-leaved forest, coniferous forest, mixed forest, natural grassland, and built grassland, based on vegetation structure. (**b**) A detailed view of green space type overlaid on a high-resolution aerial map.

Green space corresponding to forest and grassland was extracted from the Level-3 map, categorizing them into natural and built green spaces; then, we quantified the percentage of each type of green space present within administrative districts. Forests were defined as areas composed of broadleaf, coniferous, and mixed forests, with tree canopy coverage of ≥70%, meeting the conditions for forestry. Grassland was defined as an area that includes natural grasslands, created grasslands such as golf courses, cemeteries, and roadside green areas. Detailed definitions and classification criteria are presented in Table S1, and a visual representation is presented in Figure 1.

For sensitivity analysis, grasslands were further subdivided into built and natural categories based on their integration with urban infrastructure and accessibility characteristics. Built green spaces included street trees, apartment complex landscaping, neighborhood parks, golf courses, and roadside vegetation that are integrated into daily urban environments. Natural green spaces included mountainous forests and undeveloped grasslands that typically require deliberate travel to access. This classification allows examination of differential health effects based on green space accessibility and integration with residential environments.

Green space data were extracted for 73 administrative districts in 7 metropolitan cities (Seoul, Busan, Daegu, Ulsan, Daejeon, Gwangju, and Incheon) in South Korea, excluding the border areas near the Military Demarcation Line. Green space data were extracted from different years according to available Level-3 maps: Seoul, Busan, Daegu, and Ulsan in 2010; Incheon in 2012; Daejeon in 2014; and Gwangju in 2016. Previous studies have demonstrated that green space changes in urban areas tend to occur slowly, especially in compact cities with limited development space and established green infrastructure [23]. In addition, while urban land cover changes typically occur over time scales of years to decades, Seoul showed a stable land cover pattern in the majority of areas over a 32-year period (2008–2013) [24]. To assess the stability of land cover maps, we extracted Seoul green space data from the Level-3 maps for 2010 and 2018 and found no remarkable changes, with variations within ±3% (Table S2). Therefore, land cover classification from 2010 can be considered a valid proxy for exposures in the nearby years. Green space data were extracted using QGIS software, version 3.40.3.

### 2.3. Covariates

Several covariates were selected for the analysis, including season of birth, income level, unmet medical need rate, number of obstetricians and gynecologists (OB/GYN) clinics, population density, annual mean temperature, and air pollution. All covariate data were matched to the year immediately preceding the birth year. The season of birth was categorized as winter (from December to February of the following year), spring (from March to May), summer (from June to August), and fall (from September to November). Income levels were classified into low (1st to 5th deciles) and high income (6th to 9th deciles), based on income distribution by health insurance premium. Unmet medical need rate was defined as the percentage of people who were unable to visit a hospital when they wanted to in the past year within the district where they lived. Since data from 2007 and 2010 were unavailable, data from 2008 and 2011 were used instead. The number of OB/GYN clinics was calculated using the average value from 2006 and 2011 based on the status of hospital departments in administrative districts. Population density was calculated as the number of people residing in the total area of the administrative district (person/m^2^). Hourly ambient temperature (°C) data were obtained from the Korea Meteorological Administration and used as an annual average. Air pollution data were sourced from Air Korea [25], which provides hourly monitoring of air pollution concentrations in administrative districts. The air pollution metrics utilized in this study included PM_2.5_ (µg/m^3^), nitrogen dioxide (NO_2_, ppb), carbon monoxide (CO, ppb), ozone (O_3_, ppb), and sulfur dioxide (SO_2_, ppb), measured in 73 districts over the period from 2007 to 2012.

### 2.4. Statistical Analysis

Differences between cases and controls were investigated using a chi-square test, *t*-test or Mann–Whitney U test, as appropriate. and the distribution of grassland and forest for each CA type was examined. Subsequently, a generalized estimating equation (GEE) model was employed with an exchangeable correlation structure to account for the spatial clustering of residents within the same urban area. The GEE approach was chosen over traditional regression methods because it accounts for the correlation between observations within the same geographical cluster while providing population-averaged estimates of the association between green space and CA. Each CA category was analyzed separately as an independent outcome, rather than conducting simultaneous multiple comparisons. For ease of interpretation, effect estimates were expressed per 10% increase in exposure.

To account for physiological differences, sex-specific subgroup analyses were conducted, and interaction effects were tested to assess effect modification by sex. Dose–response relationships were modeled using generalized additive models (GAMs) with penalized splines to allow for potential non-linear associations. Additionally, sensitivity analyses based on exposure quartiles were performed to assess the robustness of the findings. To refine exposure characterization, the green space variables were categorized further into subtypes, allowing for a more detailed examination of their associations with the outcome.

All analyses were conducted using SAS (version 9.4; SAS Institute, Cary, NC, USA). The R software (version 4.3.0; The R Foundation, Vienna, Austria) with the package “ggplot2” was used to draw a forest plot with error bars. All statistical tests were two-sided, and *p* < 0.05 was considered statistically significant.

## 3. Results

### 3.1. Descriptive Statistics

The number of cases and prevalence per 10,000 births for each CA type is shown in Table 1. Circulatory system anomalies were the most prevalent (44.9 per 10,000 live births), and nervous system anomalies were the least prevalent (4.9 per 10,000 live births). Of the total 142,422 children, 35,629 were classified as the case group and 106,793 as the control group (Table 2). Statistically significant differences were observed between the case and control groups, with the highest proportion of cases among children born in fall (27.1%). Post hoc pairwise comparisons with Bonferroni correction revealed that children born in the fall had significantly higher than all other seasons (*p* < 0.001), while no significant difference was observed between winter and spring. The proportion of unmet medical needs and O_3_ and SO_2_ concentrations were also higher in the cases. The green space characteristics were divided by CA categories into total/grass/forest, and the distribution of each was calculated (Table S3). For the overall CA, the total percentages of total green space, grassland, and forest were 40.64%, 11.35%, and 29.29%, respectively. The amount of green space of each CA was similar to that of the overall CA.

### 3.2. Association Between Green Space and Congenital Anomalies

**Figure 2:**
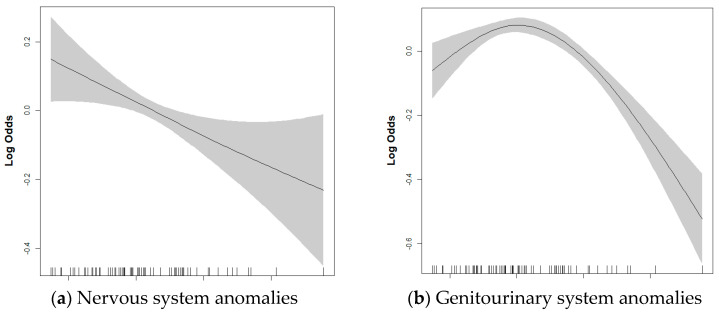
Dose–response relationship of each grassland green space with (**a**) nervous system and (**b**) genitourinary system anomalies in the full adjusted multivariable GAM. Shaded areas represent 95% confidence intervals. Models were adjusted for sex, birth year, season of birth, income, temperature, population density, unmet medical need rate, OB/GYN clinics, PM_2.5_, NO_2_, CO, SO_2_, and O_3_. OB/GYN, obstetrics and gynecology; PM_2.5_, airborne particles with an aerodynamic diameter ≤ 2.5 μm; NO_2_, nitrogen dioxide; CO, carbon monoxide; SO_2_, sulfur dioxide; O_3_, ozone.

The associations between exposure to green space and each CA are presented in Table 3. According to the results of the GEE model, total green space and forests were not associated with any CA. However, for every 10% increase in grassland, the odds of nervous system anomalies (adjusted odd ratio [aOR]: 0.77, 95% CI: 0.63–0.94) decreased by 23% and the odds of genitourinary system anomalies (aOR: 0.83, 95% CI: 0.71–0.97) decreased by 17%. While not statistically significant, a trend toward decreased odds was observed in other types of CAs as well as overall CA. Subgroup analysis by sex showed significant associations for the nervous system (aOR: 0.74, 95% CI: 0.59–0.97) and genitourinary system anomalies (aOR: 0.83, 95% CI: 0.71–0.97 only in male infants (Table S4). However, no significant interaction by sex was found. A GAM including smoothing splines with four degrees of freedom was used to visualize dose–response relationships between grassland and nervous and genitourinary system, which were identified as significant in the GEE analysis (Figure 2). For the nervous system, a linear pattern was observed, while the genitourinary system exhibited a non-linear, inverted U-shape pattern.

### 3.3. Sensitivity Analysis

To ensure the robustness of our results, we performed two sensitivity analyses. Green space exposure was divided into quartiles, and the associations related to each quartile were estimated to evaluate the trend across increasing exposure levels (Table S5). Consequently, a decrease in digestive system anomalies was observed when grassland was in the third quartile compared to in the first quartile (aOR: 0.79, 95% CI: 0.63–0.996), and genitourinary system anomalies showed a significantly negative trend (*p* for trend = 0.04). Conversely, forest demonstrated a significantly positive trend for digestive system anomalies (*p* for trend = 0.05). In addition, green spaces were divided into built and natural according to the land cover map classification system and conducted a GEE analysis in quartiles (Table S6). Built green space in the third and fourth quartiles was found to be associated with lower odds of nervous system anomalies compared to exposure to the first quartile (Q3 vs. Q1: aOR: 0.74, 95% CI: 0.56–0.98; Q4 vs. Q1: aOR: 0.71, 95% CI: 0.53–0.96), whereas only a significant trend was observed for musculoskeletal system anomalies (*p* for trend = 0.05).

## 4. Discussion

This study found an association between prenatal grassland exposure and CAs of the nervous and genitourinary systems in urban areas of South Korea. For every 10% increase in grassland coverage, significant reductions in odds were observed for nervous system (aOR: 0.77, 95% CI: 0.63–0.94) and genitourinary system (aOR: 0.83, 95% CI: 0.71–0.97) anomalies. No associations were observed for total green space or forest exposure. In the sensitivity analyses distinguishing built from natural green spaces, the association was observed only for built green spaces, with higher quartiles of built green space showing reduced odds of nervous system anomalies compared to the lowest quartile. Sex-specific analyses revealed significant associations only among male infants, although no significant interaction between greenness exposure and sex was detected.

Our study contributes to the emerging literature examining the relationship between green space exposure and congenital anomalies, which remains limited. A Chinese study found that increased green space was associated with reduced congenital heart defect risk [12], and a US study found that residential green space lowered the risk of certain structural birth defects [13]. However, both studies used NDVI without distinguishing green space types or accessibility patterns. NDVI is a useful exposure assessment index, but it has limitations such as low spatial resolution, lack of vegetation type differentiation, and negative values for water surface, which may lead to heterogeneity in the results [26]. A recent study comparing urban green space connectivity using land cover maps and NDVI data have pointed out that NDVI may oversimplify within-class variability [27]. Our study extends this limited evidence by examining different green space types and their accessibility characteristics. The finding that grassland, but not forest, showed protective associations suggests that green space accessibility and integration with daily urban environments are important factors in determining health effects [28]. This represents an important advance in our understanding of how qualitative characteristics of green space, rather than simple vegetation quantity, may influence CA risk. Further research is needed to stratify and classify the distance between housing areas and forests. The differential effects observed between grassland and forest exposure likely reflect differences in accessibility and exposure opportunities. The grassland classification in our study included street trees, apartment landscaping, neighborhood parks, and recreational areas that are typically more accessible for regular use by pregnant women than forests that are often located in remote areas.

When green spaces were further categorized into built and natural types, beneficial associations were observed only for built green spaces. This finding suggests that green spaces integrated with daily urban environments may provide more consistent exposure than remote natural areas. The accessibility and integration of green infrastructure with residential settings may be key factors determining health benefits during pregnancy [29,30,31]. These results align with recent research demonstrating that green space morphology, particularly connectivity and aggregation, may be more important for health outcomes than simple vegetation quantity. This is consistent with recent studies showing that quality and accessibility of green space may be more important determinants of health benefits than proximity alone [17,32].

Recent research has also drawn attention to how green space may reduce exposure to air pollution during pregnancy. Several birth cohort studies have reported that the beneficial effects of green space on fetal development weakened after adjusting for pollutants such as PM_2.5_ or NO_2_, implying that part of the effect may be mediated through improved air quality [33,34]. Notably, the associations between green space exposure and good birth outcomes were found to be stronger among pregnant women living in areas with lower air pollution [35], providing further support for the notion that green spaces can mitigate environmental risks. These findings indicate that the protective role of green space could extend beyond psychosocial benefits, encompassing physical environmental pathways that impact fetal development.

We found significant associations between grassland exposure and nervous and genitourinary system anomalies, but both medical and statistical research on these specific relationships remains very limited. Previous studies have shown that adverse environmental conditions during pregnancy can cause developmental and linguistic delays [36,37], hypospadias [38], and neural tube defects [39]. Green space exposure may indirectly protect fetal health by mitigating extreme heat exposure [40,41]. Further research is needed to determine the neurodevelopmental mechanisms by which maternal exposure to green space during pregnancy affects the fetus. Built green spaces, which typically offer higher accessibility in urban environments, may be particularly effective in reducing maternal stress through frequent and consistent exposure. Supporting evidence comes from studies showing that higher green space ratios and better park accessibility are associated with reduced risk of premature birth and low birth weight [42]. Access to green space during pregnancy is associated with reduced hair and cord blood cortisol concentrations, indicating reduced maternal stress levels [43,44]. These studies indicate the presence of a biological pathway through which maternal stress influences the hypothalamic–pituitary–adrenal axis [45]. Conversely, limited access to green space may lead to chronic maternal stress, which may elevate cortisol levels and interfere with normal fetal organogenesis and neurodevelopment during the critical embryonic period when fetal organ systems are most vulnerable to environmental influences [46]. Another study, which classified green space, also reported that built green space was associated with increased intelligence quotient in children [47]. These previous studies suggest the health benefits of the proximity, accessibility, frequency, and quality of exposure to nature [28,48].

The sex-specific effects observed in male fetuses warrant further investigation. While our study found significant associations only among male infants, the underlying reasons remain unclear. The absence of significant sex interactions and limited evidence for sex-specific biological mechanisms in this context suggest that these findings should be interpreted cautiously and require replication in independent studies to determine whether our result represents a true biological difference or a chance finding. Male infants and toddlers may be more active than female ones, resulting in greater exposed to outer environmental hazards and potentially difference impacts compared to females.

Overall, our findings suggest that the distribution and type of green space in urban environments can have a significant impact on prenatal health. The association between grassland area and reduced birth defect rates suggests that built environment planning can serve as an upstream intervention point for maternal and child health. Furthermore, the association between green space exposure and reduced CA observed only in male infants suggests the presence of potential sex-specific differences in vulnerability, highlighting the importance of early life interventions tailored to subgroups. These findings support the integration of green space type into public health strategies, particularly in high-density urban environments where environmental health inequalities may be exacerbated.

The strengths of our study are that, first, we utilized NHIS claims data of 142,422 participants, providing substantial statistical power to detect associations and enabling robust subgroup analyses. Second, our exposure assessment was conducted with high-resolution land cover maps that categorized specific green cover types rather than relying on vegetation indices such as NDVI, allowing for a more detailed characterization of the green environment and potential identification of differential effects across green cover categories. Third, the use of nationwide administrative health data minimized selection bias and provided comprehensive coverage of the target population, reducing concerns about generalizability within the Korean context. Finally, our study addresses an important research gap by examining the relationship between green space exposure and congenital anomalies in an Asian population, contributing valuable evidence to the growing international literature on environmental health and birth outcomes.

Despite these strengths, some limitations should be acknowledged. First, this study lacks individual-level variables for major confounding factors such as maternal age, gestational age, smoking, drinking, and nutritional status owing to limitations in NHIS claims data. This is a major limitation of this study, as it may result in residual confounding factors overestimating or underestimating the actual association. Although we adjusted for regional healthcare access using district-level unmet medical need rates, individual and regional-level variations in healthcare utilization and preventive care practices (such as folic acid supplementation) may still influence our findings [49]. Second, green space exposure was measured at the district level, which may not accurately reflect individual exposure patterns and could introduce misclassification bias. However, the high population density in Korean urban areas and the tendency for pregnant women to conduct most daily activities near their residence suggest that district-level measures may reasonably approximate individual exposure patterns. Third, we used green space data from different years (2010–2016) for births occurring in 2008–2013, assuming stable land cover patterns over time, which should be discretely measured and confirmed. Fourth, our cross-sectional design precludes causal inference, and the observed associations need to clarify unmeasured confounding factors. Fifth, the simultaneous examination of multiple congenital anomaly types may have increased the potential for chance findings owing to multiple comparisons. Sixth, the prevalence of congenital anomalies in the study population may be underestimated owing to undetected cases or fetal deaths. Finally, our control group selection approach, while innovative, has not been widely validated across different populations and healthcare systems, which may limit the generalizability of our findings.

## 5. Conclusions

To our knowledge, this study is the first to examine the relationship between green space types and congenital anomalies in urban areas of South Korea. We found that exposure to grassland, a more accessible and integrated green space, was associated with reduced risk of certain congenital anomalies. These findings highlight the potential role of urban green infrastructure in promoting maternal and child health and suggest the value of considering the inclusion of green spaces in public health planning.

## Figures and Tables

**Table 1:** Number of cases (number per 10,000 births) of congenital diseases (2008–2013).

Type of Congenital Diseases	Cases
Overall congenital diseases ^(1)^	35,629 (124)
Nervous system	1416 (4.9)
Eye, ear, nose, and face	2702 (9.4)
Circulatory system	12,893 (44.9)
Digestive system	3426 (11.9)
Genitourinary system	7398 (25.8)
Musculoskeletal system	12,123 (42.2)

^(1)^ “Overall congenital diseases” comprises children with one or more congenital anomalies, including those with multiple anomalies.

**Table 2:** Descriptive characteristics of the study population in the urban area, 2008–2013.

Exposure	Total	Case	Control	*p*-Value
	(N = 142,422)	(*n* = 35,629)	(*n* = 106,793)	
Sex, N (%)				
Male	78,334 (55.00)	19,601 (55.01)	58,733 (55.00)	0.96
Female	64,088 (45.00)	16,028 (44.99)	48,060 (45.00)	
Season of birth, N (%)				
Winter (December–February)	35,988 (25.27)	8526 (23.93)	27,462 (25.72)	**<0.0001**
Spring (March–May)	37,193 (26.11)	8748 (24.55)	28,445 (26.64)	
Summer (June–August)	33,997 (23.87)	8715 (24.46)	25,282 (23.67)	
Fall (September–November)	35,244 (24.75)	9640 (27.06)	25,604 (23.98)	
Birth year, N (%)				
2008	12,156 (6.06)	3040 (8.53)	9116 (8.54)	1.00
2009	14,031 (7.00)	3509 (9.85)	10,522 (9.85)	
2010	19,518 (9.74)	4881 (13.70)	14,637 (13.71)	
2011	27,604 (13.77)	6904 (19.38)	20,700 (19.38)	
2012	33,115 (16.52)	8286 (23.26)	24,829 (23.25)	
2013	35,998 (17.96)	9009 (25.29)	26,989 (25.27)	
Income level, N (%)				
Low	13,546 (9.51)	3385 (9.50)	10,161 (9.51)	0.94
High	128,876 (90.49)	32,244 (90.50)	96,632 (90.49)	
Unmet medical need rates, Mean (SD)	13.59 (3.48)	13.65 (3.51)	13.57 (3.47)	**0.0002**
OB/GYN clinics, N (%)	33.43 (18.05)	33.54 (18.22)	33.40 (17.98)	0.19
Population density (p/m^2^), N (%)	227.4 (446.5)	233.6 (472.5)	225.4 (437.4)	**0.0038**
Temperature, Mean (SD)	12.89 (0.94)	12.90 (0.94)	12.89 (0.94)	**0.04**
Air pollution, Median (IQR)				
PM_2.5_ (μg/m^3^)	28.82 (6.92)	28.82 (7.09)	28.82 (6.88)	0.58
NO_2_ (ppb)	28.92 (13.79)	28.92 (14.03)	28.92 (13.63)	0.06
CO (ppb)	572.98 (140.36)	572.98 (143.74)	572.98 (136.48)	0.42
SO_2_ (ppb)	5.25 (1.80)	5.22 (1.82)	5.25 (1.76)	**0.03**
O_3_ (ppb)	21.35 (5.62)	21.38 (5.73)	21.35 (5.59)	**0.004**

Note: Categorical variables represent column percentages (within-group distributions) owing to the 1:3 case–control matching design. Continuous variables represent row percentages (across-group distributions); OB/GYN, obstetrics and gynecology; PM_2.5_, airborne particles with an aerodynamic diameter ≤ 2.5 μm; NO_2_, nitrogen dioxide; CO, carbon monoxide; SO_2_, sulfur dioxide; O_3_, ozone. Bold denotes significant associations.

**Table 3:** Association between each green space type (10% increment) and CA categories from GEE models.

	Odds Ratio (95% CI)		
Type of Congenital Diseases	Total	Grassland	Forest
Overall congenital diseases ^(1)^	0.96 (0.92, 1.01)	0.88 (0.73, 1.05)	0.98 (0.94, 1.02)
Nervous system	1.00 (0.93, 1.07)	**0.77 (0.63, 0.94)**	1.02 (0.95, 1.08)
Eye, ear, nose, and face	0.97 (0.91, 1.03)	0.85 (0.71, 1.02)	0.99 (0.94, 1.04)
Circulatory system	0.96 (0.92, 1.01)	0.86 (0.70, 1.06)	0.98 (0.93, 1.02)
Digestive system	1.05 (0.98, 1.13)	0.84 (0.70, 1.01)	1.05 (0.99, 1.11)
Genitourinary system	1.00 (0.96, 1.04)	**0.83 (0.71, 0.97)**	1.01 (0.97, 1.05)
Musculoskeletal system	0.98 (0.93, 1.02)	0.88 (0.74, 1.05)	0.99 (0.95, 1.03)

^(1)^ “Overall congenital diseases” includes children with one or more congenital anomalies, including those with multiple anomalies. Note: Bold denotes significant associations; 95% CI: 95% confidential interval; all models were adjusted for sex, birth year, season of birth, income, temperature, population density, unmet medical need rate, OB/GYN clinics, PM2.5, NO_2_, CO, SO_2_, and O_3_. OB/GYN, obstetrics and gynecology; PM_2.5_, airborne particles with an aerodynamic diameter ≤ 2.5 μm; NO_2_, nitrogen dioxide; CO, carbon monoxide; SO_2_, sulfur dioxide; O_3_, ozone.

## Data Availability

The data can be accessed from NHIS’s (http://nhiss.nhis.or.kr) National Health Insurance Data Sharing Service website. Researchers can obtain data after payment of the fee if they submit a data request application and obtain approval after review by the research committee.

## Supplementary Materials

The following are available online at https://www.mdpi.com/article/10.3390/healthcare13151886/s1, Table S1: Classification criteria and definitions of forest and grassland types; Table S2: Green space coverage 2010 and 2018 in Seoul; Table S3: Distribution of green space types for CA categories; Table S4: Associations between total/grass/forest green spaces and CA stratified by sex; Table S5: Associations between total/grass/forest green spaces and CA from quartile analysis; Table S6: Associations between built/natural green spaces and CA from quartile analysis; Table S7: Distribution of green space types for CA categories; Figure S1: Study population selection flow chart.
