# Supplementary material for: Differential Effects of Green Space Typologies on Congenital Anomalies: Data from the Korean National Health Insurance Service (2008–2013)
Source: Healthcare (Basel). 2025 Aug 1;13(15):1886. doi: 10.3390/healthcare13151886 (PMC12346772; doi:10.3390/healthcare13151886)
Supplement: Supplementary file 1 [file healthcare-13-01886-s001.zip › healthcare-3731632-Supplementary.pdf]

Supplementary Materials

Table S1. Classification criteria and definitions of forest and grassland types.

<table>
<tr><th colspan="2">Vegetation Types</th><th colspan="2">Definition</th></tr>
<tr><td rowspan="3">Forest</td><td>Broad-leaved Forest</td><td>A forest in which broad-leaved trees occupy more than 75% of the total stand area.</td><td rowspan="3">Classified based on 50m × 50m land units; areas with less than 10% tree coverage are classified as “Others,” and 10–75% coverage is categorized based on dominant tree types (broad-leaved, coniferous, or mixed).</td></tr>
<tr><td>Coniferous Forest</td><td>A forest in which coniferous trees occupy more than 75% of the total stand area</td></tr>
<tr><td>Mixed Forest</td><td>A forest in which both broad-leaved and coniferous trees are mixed, and neither type exceeds 75% of the total stand area.</td></tr>
<tr><td rowspan="4">grassland</td><td>Natural Grassland</td><td colspan="2">Naturally occurring grasslands (including lowland floodplains, valley bottoms, mountainous grasslands, etc.) where natural vegetation covers more than 10% of the area. If vegetation cover is below 10%, classified as bare land; if formed artificially, classified as built grassland. When natural vegetation appears in riparian or estuarine zones, classified as wetlands or natural areas depending on dominant landform.</td></tr>
<tr><td rowspan="3">Built Grassland</td><td colspan="2">Golf Course Grassland: Grass surfaces constructed within golf courses.</td></tr>
<tr><td colspan="2">Pasture or Managed Grassland: All artificial grasslands used for grazing, landscaping, or recreational purposes.</td></tr>
<tr><td colspan="2">Other Artificial Grassland: Artificially created grasslands due to land clearing, road buffer zones, or construction site setbacks exceeding 50m. Includes rest areas, cemeteries, ski resorts, and buffer zones with turf cover. Also includes rooftop lawns or slope-protected turf zones.</td></tr>
</table>

Table S2. Green space coverage 2010 and 2018 in Seoul.

Administrative Districts of Seoul	Forest (%)			Grassland (%)		
	2010	2018	Change	2010	2018	Change
**Average**	**22.34**	**21.69**	**-0.65**	**10.89**	**11.46**	**0.57**
Jongno-gu	43.59	41.78	-1.81	8.50	8.40	-0.1
Jung-gu	11.59	11.80	0.21	8.45	8.97	0.52
Yongsan-gu	21.94	21.61	-0.33	10.22	11.72	1.5
Seongdong-gu	3.12	3.03	-0.09	13.85	16.42	2.57
Gwangjin-gu	13.90	13.68	-0.22	9.14	9.20	0.06
Dongdaemun-gu	6.90	4.69	-2.21	10.10	11.25	1.15
Jungnang-gu	19.02	18.09	-0.93	12.59	12.59	0
Seongbuk-gu	30.08	30.03	-0.05	10.55	10.42	-0.13
Gangbuk-gu	53.86	52.87	-0.99	4.48	4.88	0.4
Dobong-gu	43.37	43.22	-0.15	6.44	6.79	0.35
Nowon-gu	41.93	42.56	0.63	12.46	12.06	-0.4
Eunpyeong-gu	40.63	36.99	-3.64	5.14	6.38	1.24
Seodaemun-gu	27.54	27.36	-0.18	7.93	9.51	1.58
Mapo-gu	3.90	3.64	-0.26	17.13	18.24	1.11
Yangcheon-gu	11.43	11.41	-0.02	13.55	14.17	0.62
Gangseo-gu	8.25	7.48	-0.77	18.32	17.47	-0.85
Guro-gu	17.12	17.08	-0.04	11.08	11.63	0.55
Geumcheon-gu	22.15	20.59	-1.56	5.76	6.53	0.77
Yeongdeungpo-gu	0.19	0.19	0	14.49	14.83	0.34
Dongjak-gu	16.05	15.57	-0.48	12.83	13.58	0.75
Gwanak-gu	44.95	44.76	-0.19	4.44	4.55	0.11
Seocho-gu	40.96	38.88	-2.08	9.71	11.08	1.37
Gangnam-gu	16.56	16.06	-0.5	13.46	14.66	1.2
Songpa-gu	6.20	6.87	0.67	17.48	16.47	-1.01
Gangdong-gu	13.17	12.01	-1.16	14.05	14.67	0.62

Table S3. Distribution of green space types for CA categories

Variables	Mean	SD	Min	25th	50th	75th	Max
**Overall CA**							
Total	40.64	15.84	7.02	27.90	40.62	53.46	78.17
Grassland	11.35	4.29	3.69	8.11	10.63	13.85	23.88
Forest	29.29	17.70	0.00	13.90	27.54	43.37	68.06
**Nervous system**							
Total	39.31	15.54	7.02	27.22	36.03	50.67	78.17
Grassland	11.31	4.17	3.69	8.25	10.72	13.85	23.88
Forest	28.00	17.41	0.00	13.89	23.89	41.93	68.06
**Eye, ear, nose, and face**							
Total	39.73	15.61	7.02	21.03	36.56	50.67	78.17
Grassland	11.38	4.14	3.69	8.25	10.72	13.85	23.88
Forest	28.35	17.41	0.00	13.89	26.90	41.93	68.06
**Circulatory system**							
Total	41.07	15.81	7.02	28.21	40.62	54.30	78.17
Grassland	11.33	4.35	3.69	8.11	10.55	14.05	23.88
Forest	29.74	17.73	0.00	13.90	30.08	43.37	68.06
**Digestive system**							
Total	40.34	15.95	7.02	27.22	36.94	54.30	78.17
Grassland	11.50	4.22	3.69	8.25	11.08	14.05	23.88
Forest	28.85	17.69	0.00	13.89	26.90	43.11	68.06
**Genitourinary system**							
Total	41.20	15.81	7.02	28.21	40.62	54.30	78.17
Grassland	11.58	4.37	3.69	8.25	11.08	14.49	23.88
Forest	29.62	17.61	0.00	13.90	30.08	43.37	54.71
**Musculoskeletal system**							
Total	40.16	15.80	7.02	27.22	36.56	51.22	78.17
Grassland	11.33	4.24	3.69	8.25	10.63	13.55	23.88
Forest	28.83	17.69	0.00	13.89	26.90	43.11	68.06

Table S4. Associations between total/grass/forest green spaces and CA stratified by sex.

	Type of congenital diseases	Boy	Girl	Interaction p-value
Total	Overall congenital diseases	0.98(0.94,1.02)	0.97(0.93,1.02)	0.98
	Nervous system	1.01(0.93,1.09)	1.02(0.94,1.11)	0.81
	Eye, ear, nose, and face	0.96(0.90,1.04)	1.00(0.93,1.06)	0.86
	Circulatory system	0.97(0.93,1.02)	0.98(0.93,1.04)	0.80
	Digestive system	1.04(0.96,1.13)	1.05(0.96,1.15)	0.98
	Genitourinary system	1.00(0.96,1.05)	1.03(0.97,1.10)	0.79
	Musculoskeletal system	0.99(0.94,1.04)	0.98(0.93,1.03)	0.67
Grassland	Overall congenital diseases	0.88(0.74,1.05)	0.88(0.74,1.06)	0.83
	Nervous system	**0.74(0.57,0.97)**	0.82(0.65,1.03)	0.31
	Eye, ear, nose, and face	0.88(0.74,1.05)	0.83(0.67,1.03)	0.86
	Circulatory system	0.88(0.73,1.06)	0.87(0.70,1.08)	0.97
	Digestive system	0.92(0.76,1.12)	0.78(0.60,1.03)	0.62
	Genitourinary system	**0.83(0.71,0.97)**	0.87(0.68,1.10)	0.61
	Musculoskeletal system	0.86(0.71,1.03)	0.92(0.78,1.09)	0.66
Forest	Overall congenital diseases	0.99(0.95,1.03)	0.98(0.94,1.03)	0.98
	Nervous system	1.03(0.95,1.10)	1.03(0.96,1.11)	0.96
	Eye, ear, nose, and face	0.98(0.92,1.04)	1.01(0.95,1.07)	0.84
	Circulatory system	0.99(0.95,1.03)	0.99(0.94,1.05)	0.99
	Digestive system	1.04(0.97,1.11)	1.06(0.97,1.15)	0.93
	Genitourinary system	1.02(0.98,1.06)	1.04(0.98,1.10)	0.91
	Musculoskeletal system	1.00(0.96,1.05)	0.99(0.95,1.03)	0.61

All models were adjusted for birth year, seasons of birth, income, temperature, population density, unmet medical needs rate, OB/GYN clinics, $PM_{2.5}$, NO_2, CO, SO_2, and O_3

Table S5. Associations between total/grass/forest green spaces and CA from quartile analysis

Type of green space		Adjusted Odds Ratio (95% CI)						
		Overall	Nervous system	Eye, ear, nose, and face	Circulatory system	Digestive system	Genitourinary system	Musculoskeletal system
Total								
	Q1	reference	reference	reference	reference	reference	reference	reference
	Q2	0.96(0.79,1.17)	1.06(0.8,1.40)	0.96(0.78,1.18)	0.98(0.77,1.25)	0.98(0.76,1.28)	0.94(0.78,1.13)	0.95(0.79,1.15)
	Q3	0.95(0.80,1.12)	1.21(0.93,1.57)	1.11(0.90,1.37)	0.98(0.81,1.19)	1.14(0.94,1.39)	0.93(0.79,1.08)	0.90(0.73,1.10)
	Q4	0.89(0.74,1.07)	1.00(0.74,1.34)	0.98(0.76,1.25)	0.87(0.70,1.08)	1.12(0.85,1.50)	1.03(0.86,1.22)	0.96(0.8,1.15)
P trend		0.24	0.94	0.95	0.21	0.28	0.86	0.62
Grassland								
	Q1	reference	reference	reference	reference	reference	reference	reference
	Q2	1.12(0.95,1.32)	0.96(0.74,1.24)	0.95(0.78,1.17)	1.10(0.91,1.32)	1.09(0.85,1.40)	1.08(0.93,1.25)	1.06(0.88,1.26)
	Q3	0.97(0.81,1.16)	0.85(0.63,1.16)	0.92(0.76,1.12)	0.96(0.78,1.18)	**0.79(0.63,0.996)**	0.90(0.76,1.07)	1.03(0.81,1.03)
	Q4	0.9(0.72,1.14)	0.78(0.58,1.04)	0.81(0.64,1.03)	0.9(0.68,1.20)	0.89(0.70,1.14)	0.84(0.69,1.02)	0.88(0.70,1.09)
P trend		0.30	0.08	0.08	0.38	0.12	**0.04**	0.32
Forest								
	Q1	reference	reference	reference	reference	reference	reference	reference
	Q2	1.01(0.84,1.22)	0.98(0.77,1.25)	1.00(0.81,1.25)	1.13(0.92,1.40)	0.96(0.79,1.18)	1.04(0.87,1.24)	0.97(0.80,1.19)
	Q3	1.01(0.82,1.23)	1.30(0.94,1.81)	1.08(0.86,1.35)	1.05(0.85,1.30)	1.18(0.91,1.53)	0.99(0.84,1.17)	0.93(0.77,1.14)
	Q4	0.97(0.81,1.15)	1.18(0.87,1.60)	1.07(0.83,1.37)	0.99(0.81,1.21)	1.24(0.96,1.61)	1.11(0.93,1.31)	1.02(0.86,1.22)
P trend		0.68	0.15	0.52	0.66	**0.05**	0.32	0.86

Note: Quartile groups are defined based on the 25th, 50th, and 75th percentiles of each green space exposure variable (see Table S3 for specific cut-off values); Bold denotes significant associations.

All models were adjusted for sex, birth year, seasons of birth, income, temperature, population density, unmet medical needs rate, OB/GYN clinics, $PM_{2.5}$, NO_2, CO, SO_2, and O_3

Table S6. Associations between built/natural green spaces and CA from quartile analysis

Type of green space	Adjusted Odds Ratio (95% CI)						
	Overall	Nervous system Circulatory system	Eye, ear, nose, and face	Circulatory system	Digestive system	Genitourinary system	Musculoskeletal system
Built							
Q1	reference	reference	reference	reference	reference	reference	reference
Q2	**1.25(1.04,1.49)**	0.82(0.63,1.06)	1.03(0.83,1.28)	1.18(0.98,1.43)	0.91(0.71,1.17)	1.08(0.91,1.26)	1.22(1.00,1.49)
Q3	1.05(0.87,1.26)	**0.74(0.56,0.98)**	0.84(0.68,1.04)	0.98(0.79,1.22)	0.78(0.58,1.03)	0.94(0.77,1.15)	1.03(0.85,1.26)
Q4	0.95(0.74,1.22)	**0.71(0.53,0.96)**	0.90(0.70,1.17)	0.91(0.67,1.24)	0.83(0.63,1.08)	0.84(0.69,1.04)	0.96(0.77,1.21)
P trend	0.16	0.21	0.95	0.08	0.41	0.22	**0.045**
Natural							
Q1	reference	reference	reference	reference	reference	reference	reference
Q2	1.04(0.86,1.26)	0.88(0.70,1.10)	0.97(0.78,1.20)	1.19(0.97,1.47)	1.03(0.80,1.33)	1.07(0.90,1.27)	0.95(0.78,1.15)
Q3	0.95(0.78,1.15)	1.26(0.95,1.68)	1.03(0.82,1.30)	0.99(0.80,1.21)	1.09(0.89,1.34)	0.95(0.81,1.13)	0.86(0.70,1.06)
Q4	0.96(0.81,1.13)	1.09(0.83,1.44)	1.06(0.84,1.34)	1.00(0.82,1.22)	1.19(0.92,1.55)	1.11(0.93,1.31)	0.99(0.82,1.18)
P trend	0.73	0.51	0.73	0.15	0.98	0.87	0.36

Note: Quartile groups are defined based on the 25th, 50th, and 75th percentiles of each green space exposure variable (see Table S7 for specific cut-off values); Bold denotes significant associations.

All models were adjusted for sex, birth year, seasons of birth, income, temperature, population density, unmet medical needs rate, OB/GYN clinics, $PM_{2.5}$, NO_2, CO, SO_2, and O_3

Table S7. Distribution of green space types for CA categories

Variables	Mean	SD	Min	25th	50th	75th	Max
**Overall CA**							
Built	9.73	4.02	2.59	7.08	9.11	11.98	20.39
Natural	30.91	17.58	0.00	15.44	30.51	44.00	71.85
**Nervous system**							
Built	9.75	3.91	2.59	7.26	9.24	11.98	20.39
Natural	29.56	17.26	0.00	14.81	28.27	43.16	71.85
**Eye, ear, nose, and face**							
Built	9.83	3.91	2.59	7.30	9.24	11.98	20.39
Natural	29.90	17.29	0.00	14.81	29.04	43.16	71.85
**Circulatory system**							
Built	9.76	4.05	2.59	7.08	8.95	11.98	20.39
Natural	31.31	17.53	0.00	15.44	30.51	44.00	71.85
**Digestive system**							
Built	10.01	4.00	2.59	7.30	9.24	12.66	20.39
Natural	30.33	17.55	0.00	14.81	29.51	43.16	71.85
**Genitourinary system**							
Built	10.01	4.18	2.59	7.09	9.24	12.66	20.39
Natural	31.18	17.48	0.00	15.44	30.51	44.00	71.85
**Musculoskeletal system**							
Built	9.61	3.93	2.59	7.08	9.11	11.81	20.39
Natural	30.55	17.62	0.00	15.44	29.51	43.16	71.85

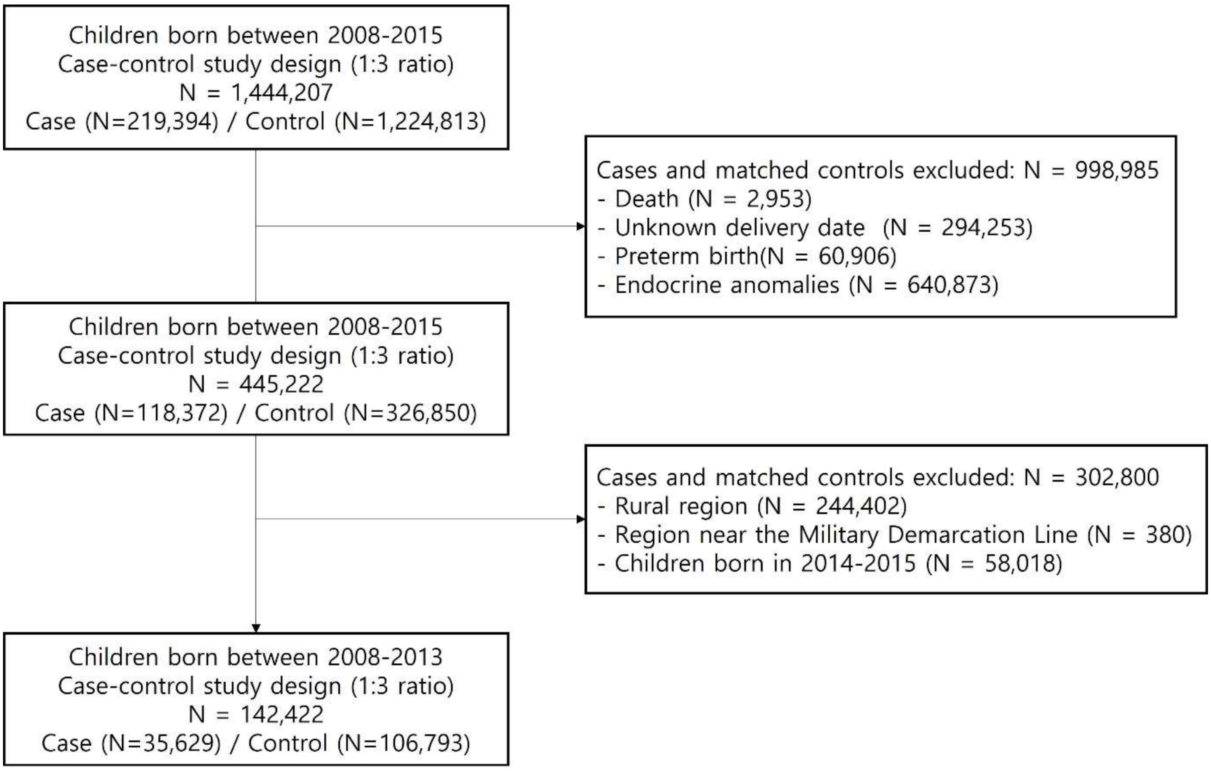

Figure S1. Study population selection flow chart
